# Supplementary material for: Reducing Effect of Farnesylquinone on Lipid Mass in C. elegans by Modulating Lipid Metabolism
Source: Mar Drugs. 2019 Jun 5;17(6):336. doi: 10.3390/md17060336 (PMC6627328; doi:10.3390/md17060336)
Supplement: Supplementary file 1 [file marinedrugs-17-00336-s001.pdf]

Reducing effect of farnesylquinone on lipid mass in *C. elegans* by modulating lipid metabolism

Supporting Information

Figure S1. ^{1}H NMR spectrum of 1 in $CDCl_3$

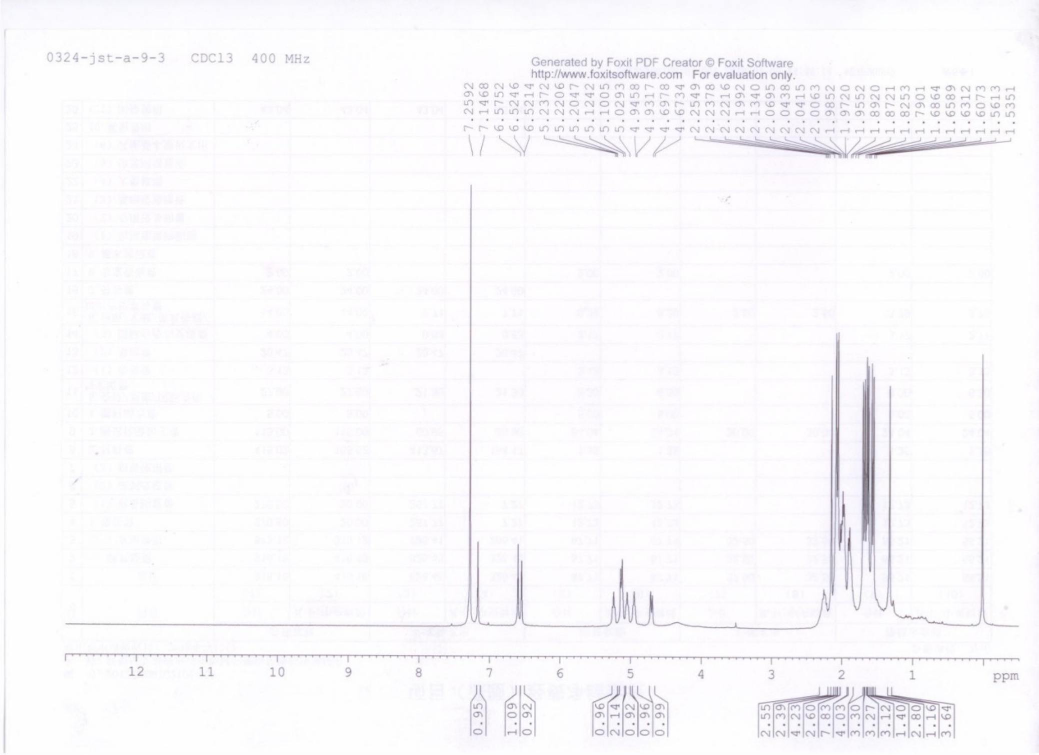

Figure S2. ^{13}C NMR (DEPT) spectrum of 1 in $CDCl_3$

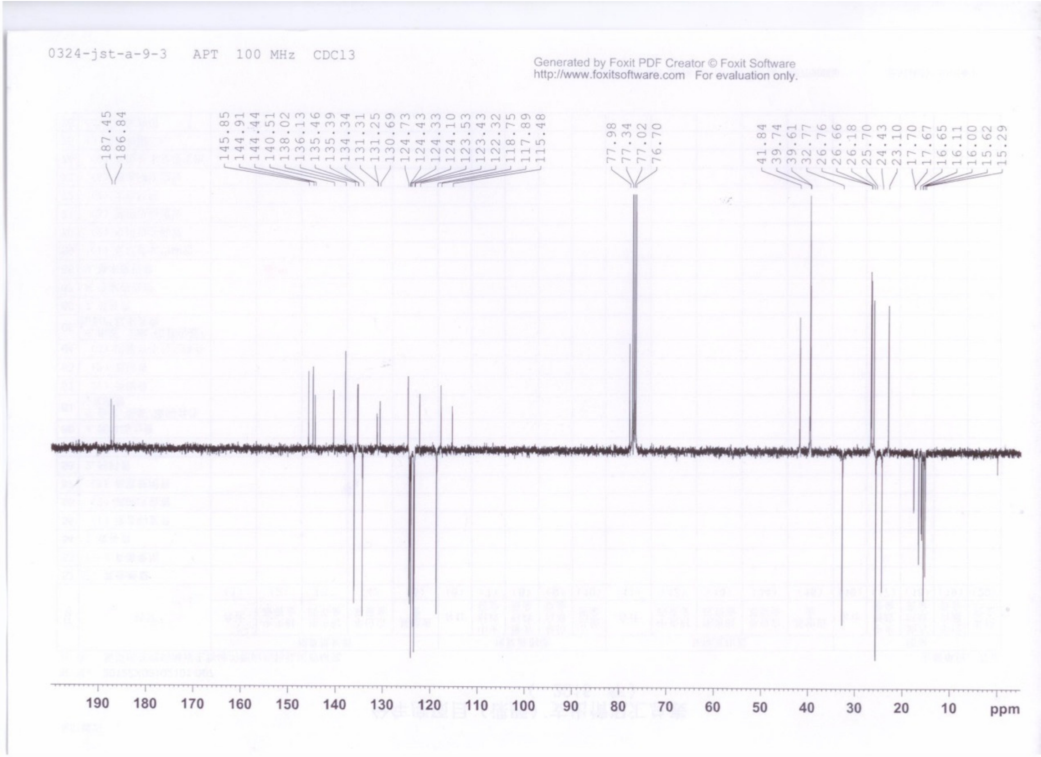

Figure S3. COSY spectrum of 1 in CDCl3

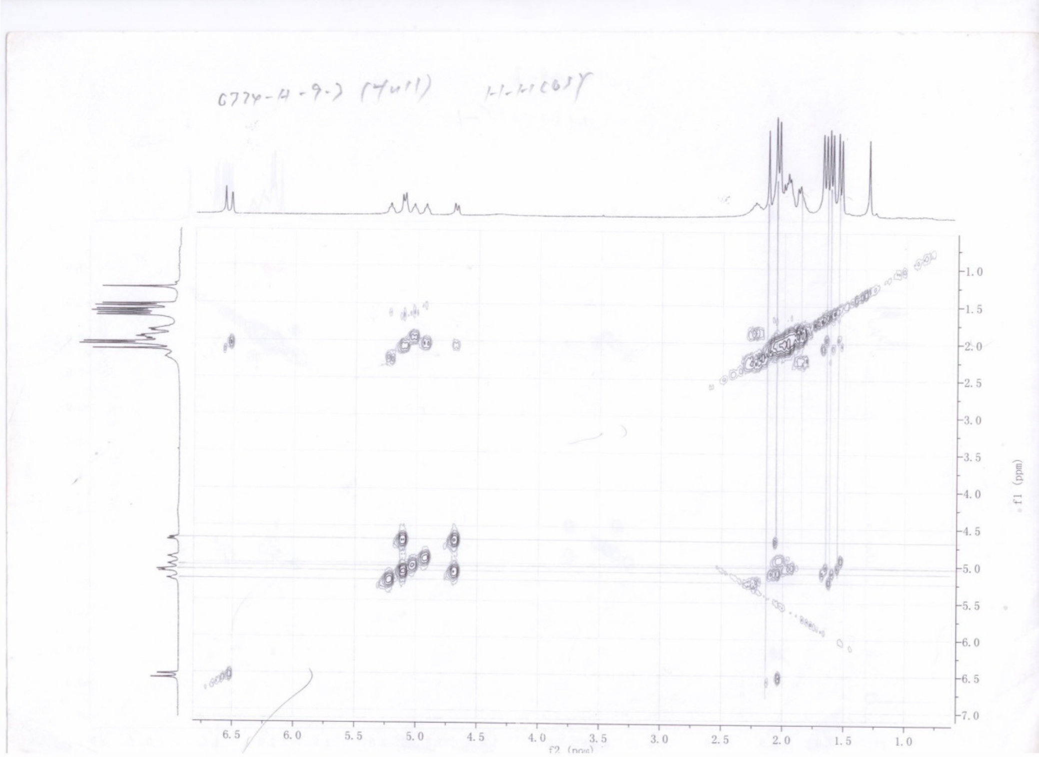

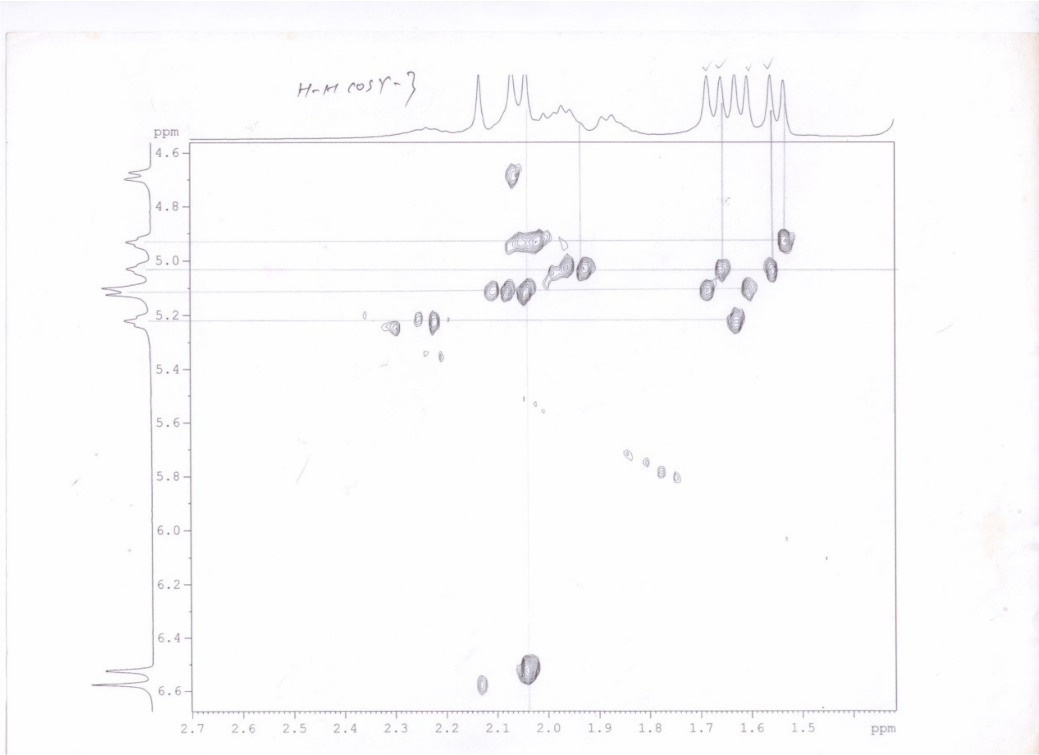

Figure S4. HSQC spectrum of 1 in $CDCl_3$

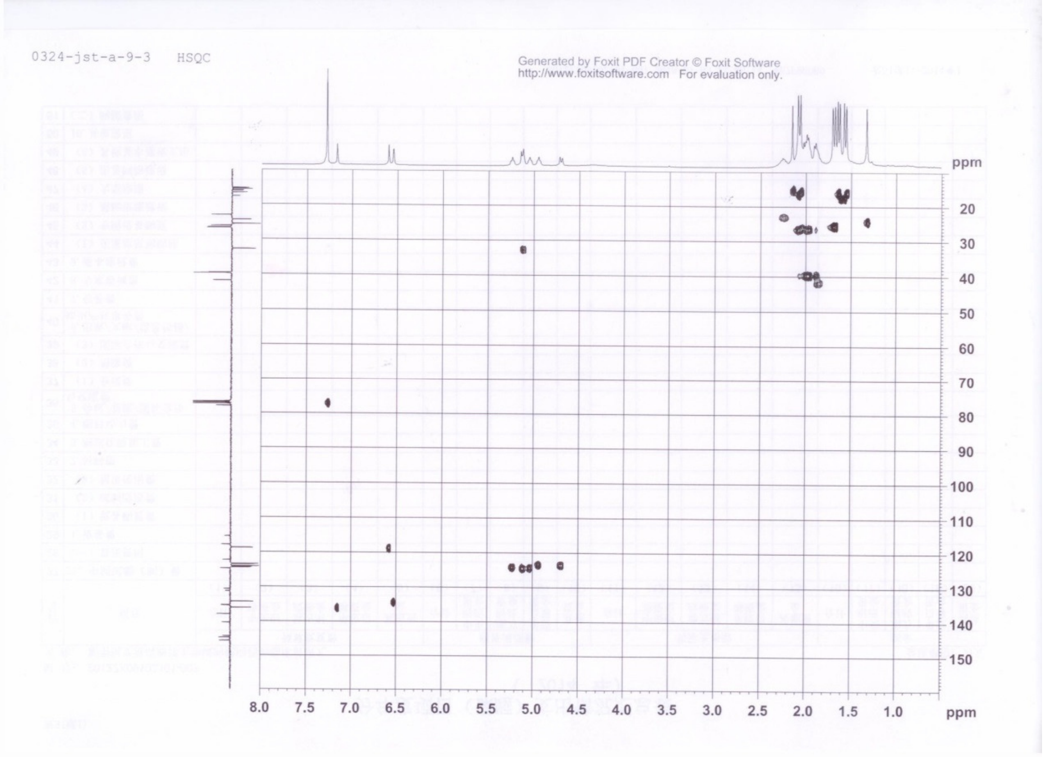

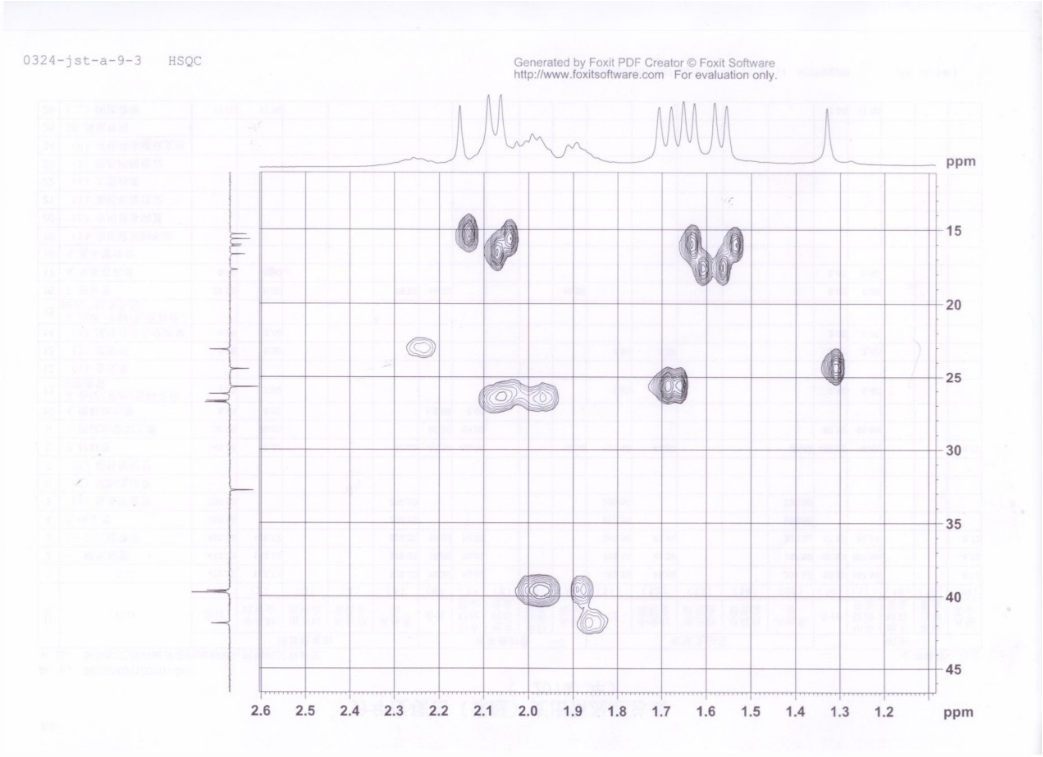

Figure S5. HMBC spectrum of 1 in CDCl3

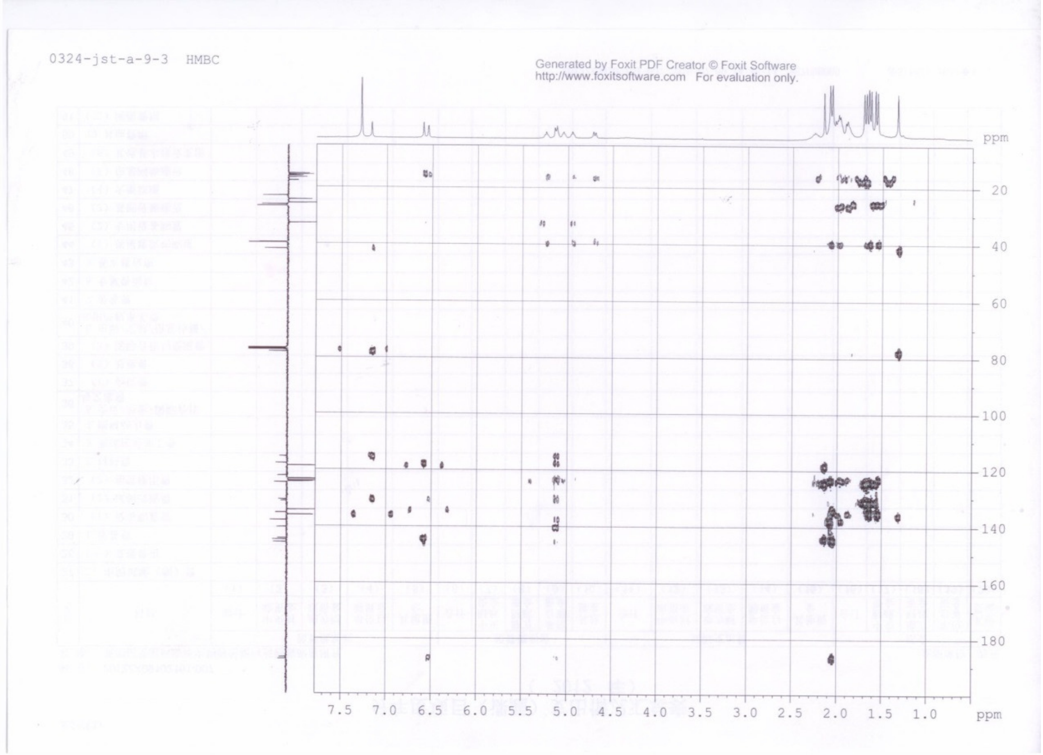

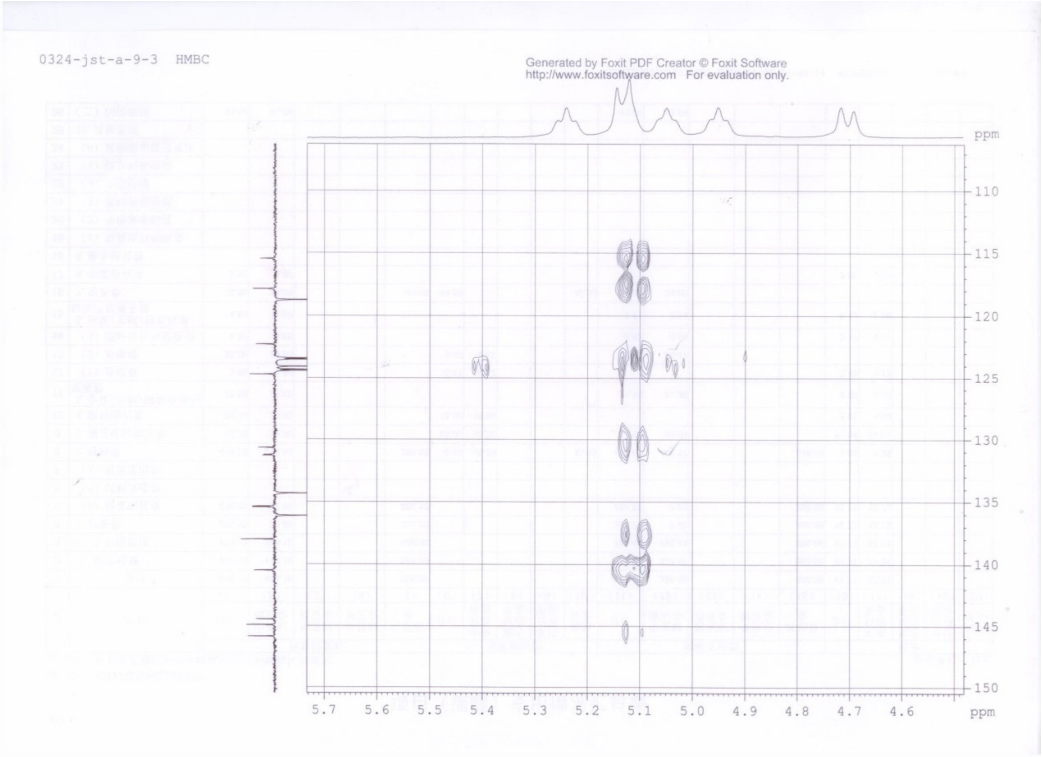

Figure S6. NOESY spectrum of 1 in CDCl3

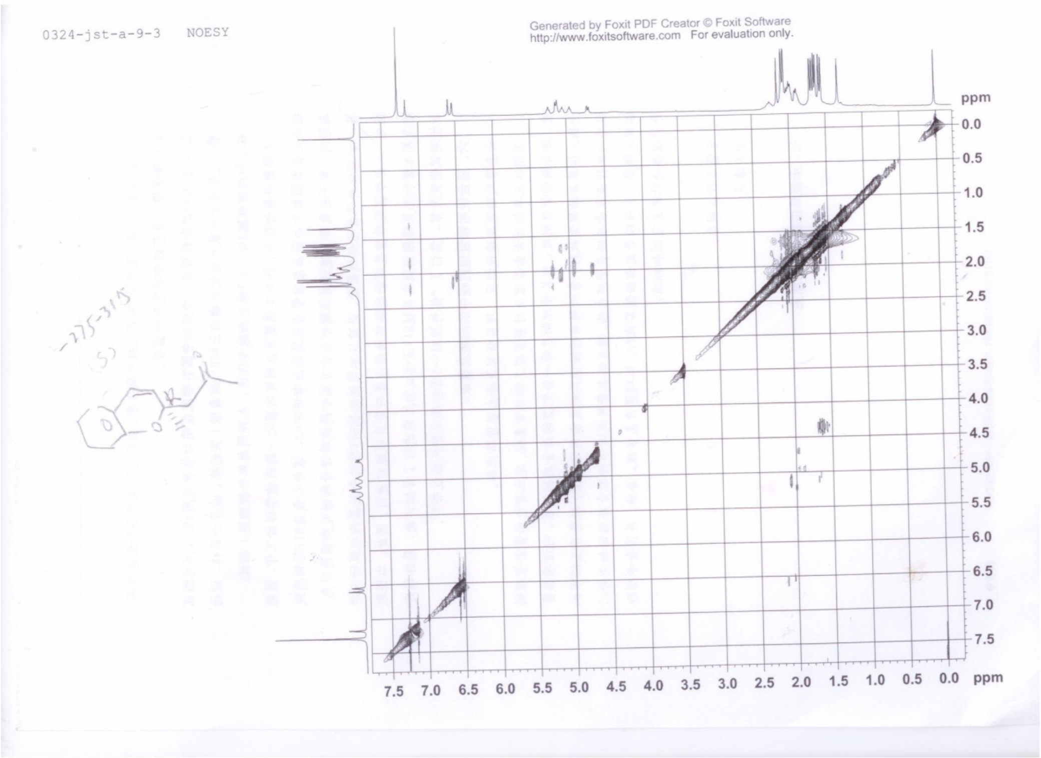

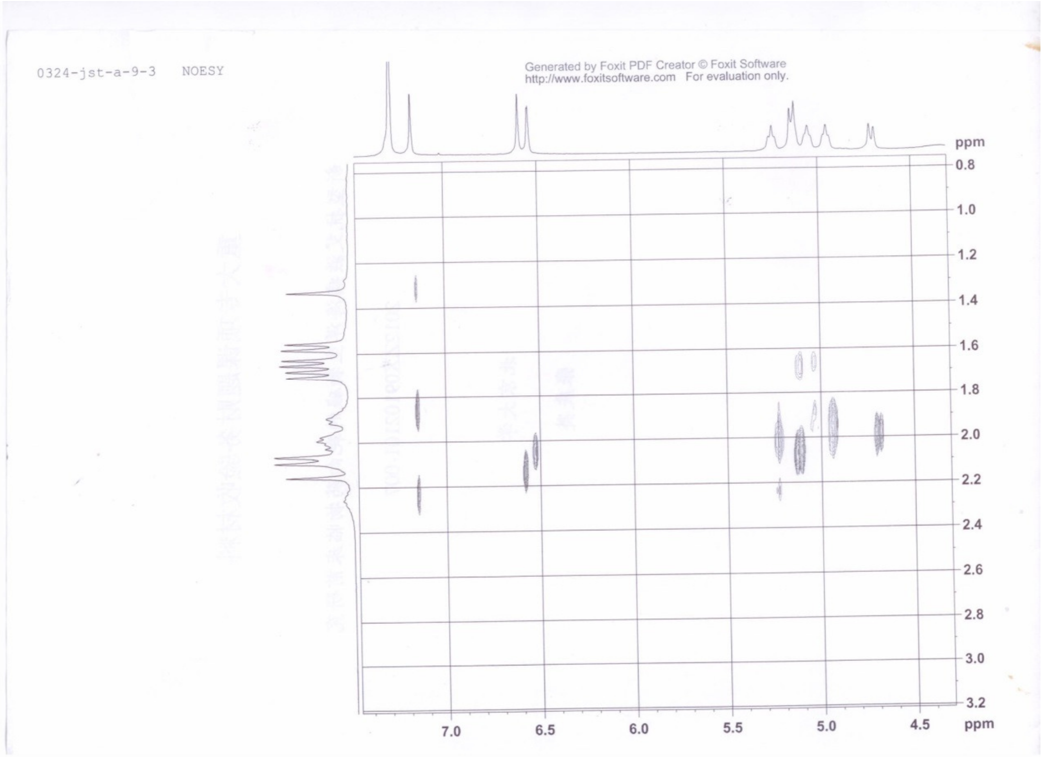

Figure S7. IR spectrum of 1

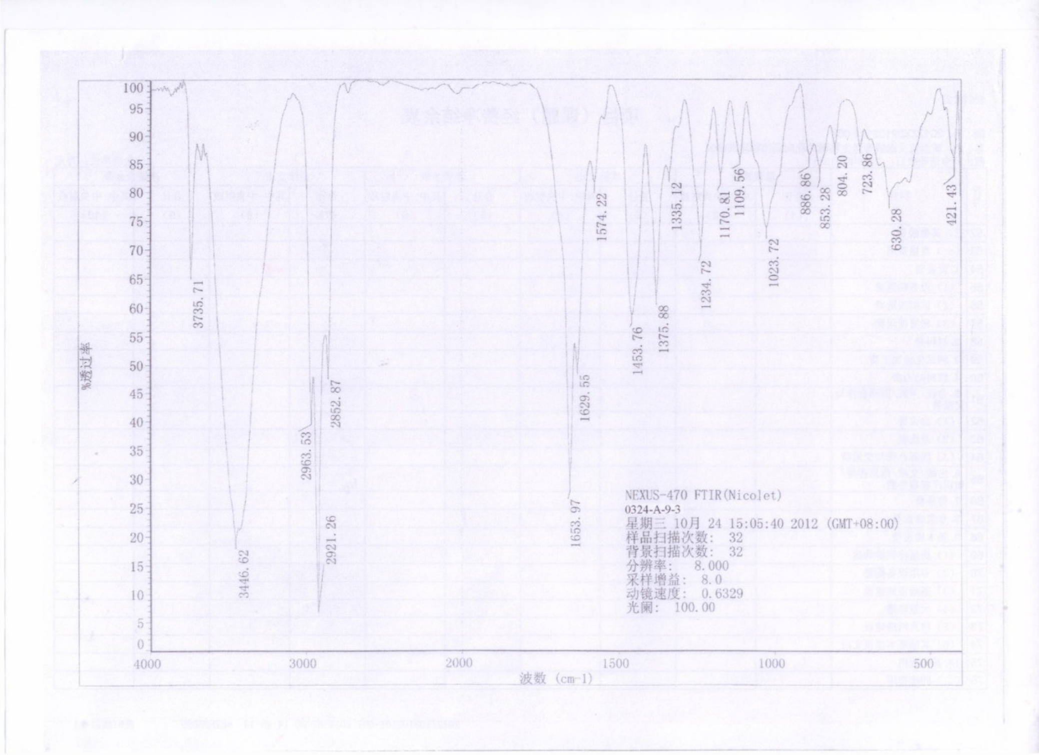

Figure S8. HRESIMS spectrum of 1

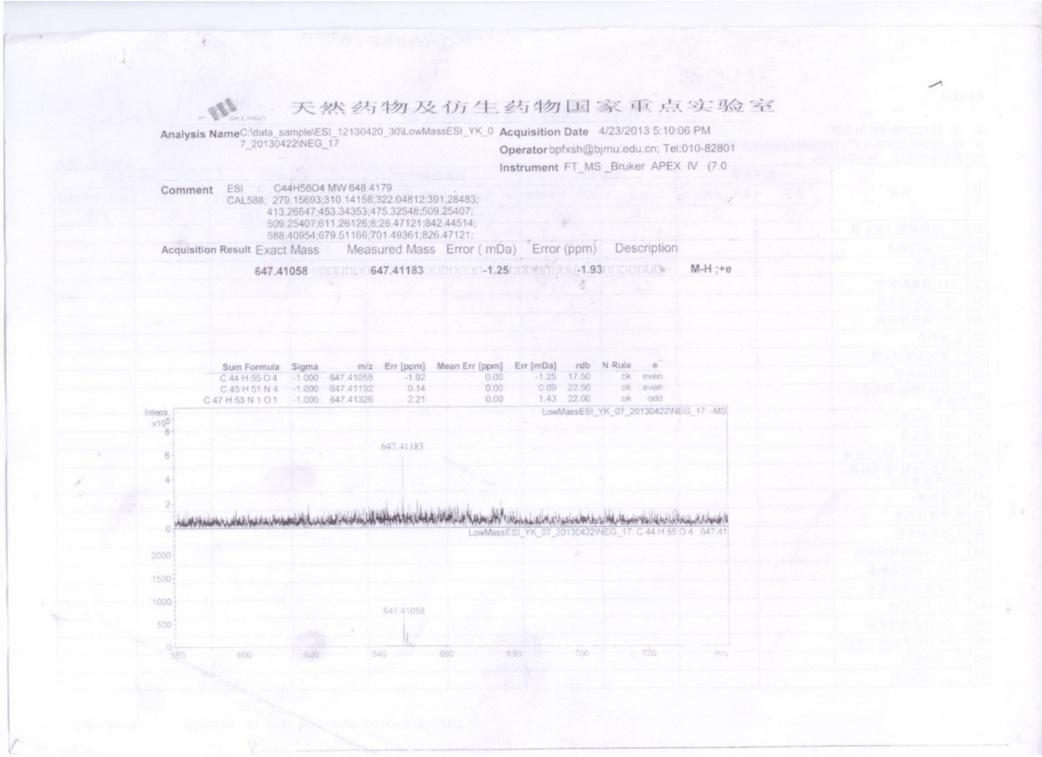

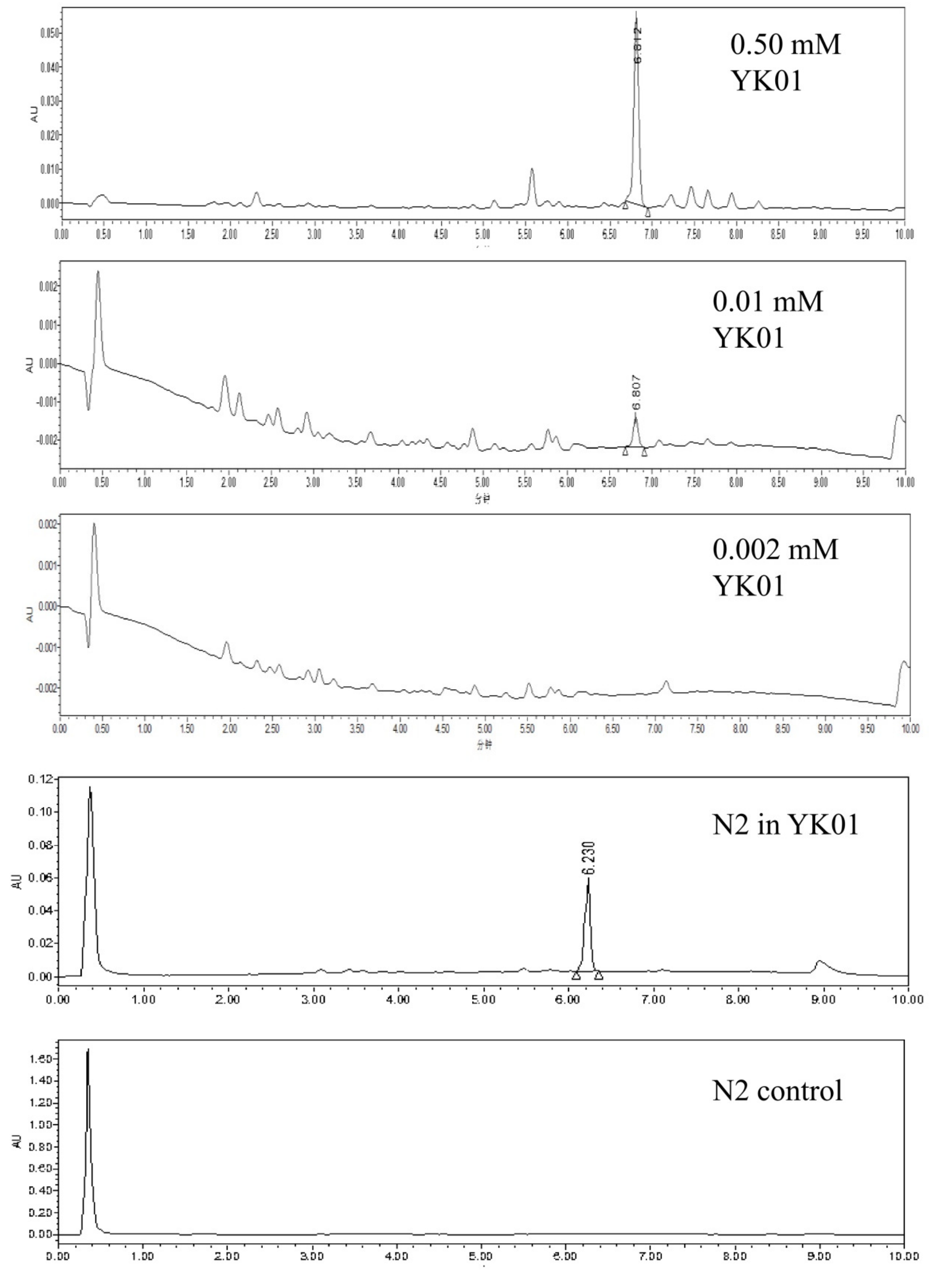

Figure S9. Measurement of the amount of ingested **2** in animals by UPLC. A. Chromatograms of YBH10-5 standards of 0.5 mM, 0.01mM and 0.002 mM concentration respectively, 0.002 mM was out the detection limit. B. 2X10^5 L4 N2 worms were put into 0.5 mM of **2** solution, methanol extract was for UPLC analysis (Upper panel); Drug soaked animals (lower panel).

Compound 2 = YKo1
